# Oncogene Mutations, Copy Number Gains and Mutant Allele Specific Imbalance (MASI) Frequently Occur Together in Tumor Cells

**DOI:** 10.1371/journal.pone.0007464

**Published:** 2009-10-14

**Authors:** Junichi Soh, Naoki Okumura, William W. Lockwood, Hiromasa Yamamoto, Hisayuki Shigematsu, Wei Zhang, Raj Chari, David S. Shames, Ximing Tang, Calum MacAulay, Marileila Varella-Garcia, Tõnu Vooder, Ignacio I. Wistuba, Stephen Lam, Rolf Brekken, Shinichi Toyooka, John D. Minna, Wan L. Lam, Adi F. Gazdar

**Affiliations:** 1 Hamon Center for Therapeutic Oncology Research, University of Texas Southwestern Medical Center, Dallas, Texas, United States of America; 2 Department of Pathology, University of Texas Southwestern Medical Center, Dallas, Texas, United States of America; 3 Department of Internal Medicine, University of Texas Southwestern Medical Center, Dallas, Texas, United States of America; 4 Department of Pharmacology, University of Texas Southwestern Medical Center, Dallas, Texas, United States of America; 5 Department of Cancer and Thoracic Surgery, Graduate School of Medicine, Dentistry and Pharmaceutical Sciences, Okayama University, Okayama, Japan; 6 Departments of Cancer Genetics and Developmental Biology, and Cancer Image, British Columbia Cancer Research Centre, Vancouver, British Columbia, Canada; 7 Department of Thoracic/Head and Neck, University of Texas MD Anderson Cancer Center, Houston, Texas, United States of America; 8 Department of Pathology, University of Texas MD Anderson Cancer Center, Houston, Texas, United States of America; 9 Department of Medicine, University of Colorado Health Sciences Center, Aurora, Colorado, United States of America; 10 Department of Biotechnology, Institute of Molecular and Cell Biology, Tartu University Hospital, Tartu University, Tartu, Estonia; 11 Oncology Diagnostics, Genentech Inc., South San Francisco, California, United States of America; The University of Hong Kong, Hong Kong

## Abstract

**Background:**

Activating mutations in one allele of an oncogene (heterozygous mutations) are widely believed to be sufficient for tumorigenesis. However, mutant allele specific imbalance (MASI) has been observed in tumors and cell lines harboring mutations of oncogenes.

**Methodology/Principal Findings:**

We determined 1) mutational status, 2) copy number gains (CNGs) and 3) relative ratio between mutant and wild type alleles of *KRAS*, *BRAF*, *PIK3CA* and *EGFR* genes by direct sequencing and quantitative PCR assay in over 400 human tumors, cell lines, and xenografts of lung, colorectal, and pancreatic cancers. Examination of a public database indicated that homozygous mutations of five oncogenes were frequent (20%) in 833 cell lines of 12 tumor types. Our data indicated two major forms of MASI: 1) MASI with CNG, either complete or partial; and 2) MASI without CNG (uniparental disomy; UPD), due to complete loss of wild type allele. MASI was a frequent event in mutant *EGFR* (75%) and was due mainly to CNGs, while MASI, also frequent in mutant *KRAS* (58%), was mainly due to UPD. Mutant: wild type allelic ratios at the genomic level were precisely maintained after transcription. *KRAS* mutations or CNGs were significantly associated with increased ras GTPase activity, as measured by ELISA, and the two molecular changes were synergistic. Of 237 lung adenocarcinoma tumors, the small number with both *KRAS* mutation and CNG were associated with shortened survival.

**Conclusions:**

MASI is frequently present in mutant *EGFR* and *KRAS* tumor cells, and is associated with increased mutant allele transcription and gene activity. The frequent finding of mutations, CNGs and MASI occurring together in tumor cells indicates that these three genetic alterations, acting together, may have a greater role in the development or maintenance of the malignant phenotype than any individual alteration.

## Introduction

**Figure 1:**
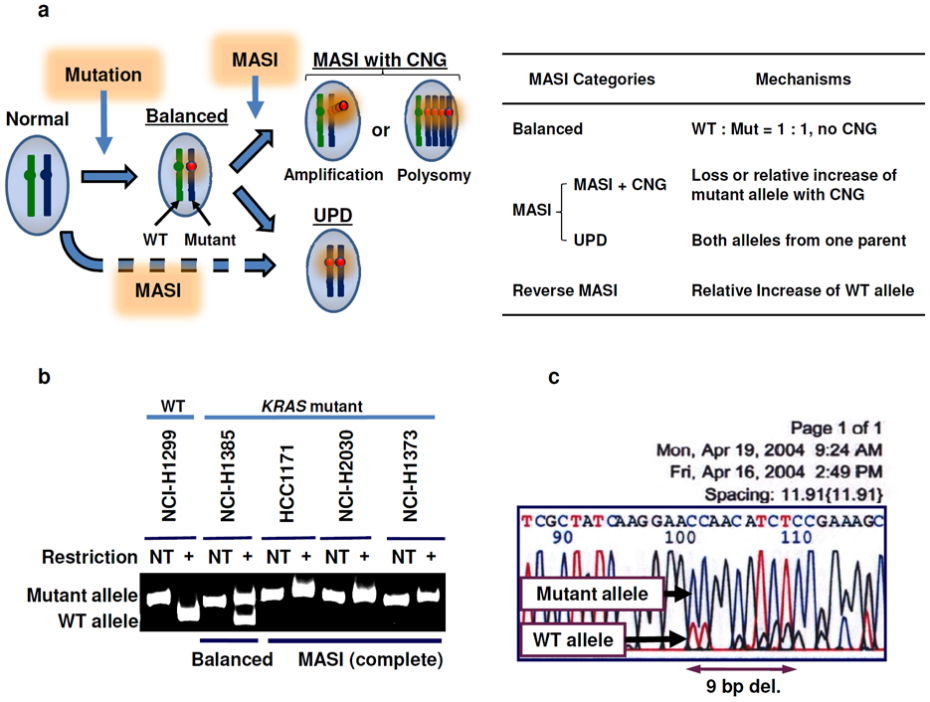
Mutant allele specific imbalance (MASI) and some earlier observations. a) types of MASI. Three major types of MASI may occur. b) Complete MASI of *KRAS* gene as identified in 1991. We reported *KRAS* mutations in non-small-cell lung cancer (NSCLC) cell lines using restriction fragment length polymorphism (RFLP) method which can digest only wild type (WT) allele. We made this figure using modified methodologies from the original publication [3]. Three out of four *KRAS* mutant NSCLC lines showed homozygous mutations (complete MASI) of *KRAS* codon 12. NT, no treatment of restriction enzyme; +, presence of treatment of restriction enzyme. c) Our first *EGFR* mutation (exon 19 deletion) showed that the mutant allele was in great excess compared to the WT allele. WT, wild type.

Oncogenes may be activated by mutation, structural rearrangement or gene copy number gains (CNGs) [1], [2]. While activating somatic mutations in one allele of an oncogene (heterozygous mutation, “one hit”) is generally believed to be sufficient to confer a selective growth advantage on the cell [1], mutant allele specific imbalance (MASI, Fig. 1a) has been observed in tumors and cell lines harboring oncogenic mutations. As early as 1991, we reported that *KRAS* mutations in cancer cell lines frequently demonstrated complete or relative MASI [3] (Fig. 1b). In April 2004 just before the two initial major publications about activating mutations of epidermal growth factor receptor (EGFR) gene appeared[4], [5], we examined a never smoker female with adenocarcinoma of the lung, and found a nine base pair deletion mutation in exon 19 of the *EGFR* gene (Fig. 1c). Even though the tumor had not been microdissected, the mutant allele appeared to be in great excess. More recently we noted the frequent presence of CNGs in tumor cells having mutant forms of the same genes [6].

Recent genome-wide approaches, especially high resolution single nucleotide polymorphism (SNP) arrays, enable evaluation of dynamic chromosomal as well as focal changes of CNG and loss of heterogeneity (LOH) with very high resolution. Within a few years, these assays have identified several novel lesions with amplification and/or LOH across several organs [7]. An important identification by SNP array was that uniparental disomy (UPD), which was originally described as a constitutional mechanism during meiosis [8], was frequently observed in several cancers [9], [10], [11], [12]. UPD arises when an individual inherits two copies of a particular chromosome from the same parent [8]. The acquisition of UPD results in homozygosity for preexisting gene mutations with selective retention of the mutated allele. Acquired UPD in association with oncogenic mutations has been reported in hematopoietic malignancies including *FLT3* and *WT1* mutations in acute myeloid leukemia [10], [13] and *JAK2* mutations in myeloproliferative disorders [14], [15]. To date, all reports of acquired UPD in solid tumors have been in association with the “two hit” inactivation of tumor suppressor genes [9], [11], [16].

*EGFR* pathway genes, including, *EGFR*, *KRAS*, *BRAF*, and *PIK3CA* genes, are well-investigated oncogenes in many tumors including lung, colorectal (CRC), and pancreatic cancers (PAC) [6], [17], [18], [19]. Activating *RAS* mutations, including *KRAS*, are the most frequent oncogenic mutations present in human tumors, detected in about 20% of non-small-cell lung cancer (NSCLC), 40% of CRC and over 90% of PAC [19]. *BRAF* and *PIK3CA* genes are also activated by mutations in CRC [17], [18], [20] and occasionally in lung cancers [21], [22]. Activating mutations of *EGFR* gene are present in 15–30% of NSCLC while they have been rarely detected in other type of human cancers [23], [24]. *EGFR* CNGs were also reported in NSCLC and may play a role in response and survival to tyrosine kinase inhibitor therapy [6], [25], [26] while *KRAS* CNGs have not been investigated in depth in clinical tumors including NSCLCs. Taken together, the inter-relationship between mutations, CNGs and MASI is complex. The goal of the present study is to better understand the complex inter-relationships between mutations, CNGs and MASI, and to clarify the biological and clinical significance of these oncogenic alterations.

## Materials and Methods

### Frequency of homozygous mutation from the Sanger Institute public database

We queried the zygosity status of 11 well-known and frequently mutated genes including six tumor suppressor genes (*TP53*, *CDKN2A*, *PTEN*, *RB1*, *APC*, and *SMAD*) and five oncogenes (*KRAS*, *BRAF*, *PIK3CA*, *NRAS*, and *EGFR*) tested in 833 cell lines from the database of the Cancer Genome Project, Sanger Institute, Cambridge, UK (www.sanger.ac.uk). We limited our examination to genes having relatively large numbers of mutations (>30) but also included the *EGFR* gene (7 mutations) which forms the basis of much of our work. Because of stromal cell contamination in clinical tumor samples, we limited our examination to tumor cell lines. We downloaded the free database of mutational status and zygosity status for each gene (on April 8^th^ 2009). Zygosity status of each mutation was determined at the Institute by manual examination of the sequencing electropherograms (response to our query, Sanger #80248). We calculated the frequency of homozygosity for each of the 11 genes and for the entire oncogene or tumor suppressor groups.

### Cell lines

We studied 114 tumor cell lines of lung cancer (n = 85), CRC (n = 19) or PAC (n = 10) origin. The details of each line are shown in Table S1. The origins of the lung lines have already been described [6], [22]. We also investigated six human bronchial epithelial cell lines (HBEC lines 2KT, 3KT, 5KT, 15KT, 17KT, and 21KT), which were initiated by us [27], [28]. All CRC and PAC lines were purchased from the American Type Culture Collection (ATCC, Manassas, VA).

All cell lines were proven to have individual genetic origins by the Powerplex 1.2 system (Promega, Madison, WI) and, when available, corresponded with their original profiles as obtained from the ATCC.

### Tumor Samples

We studied 393 tumors of NSCLC (n = 333) or CRC (n = 60) origin (Table S2a–d). DNAs from 269 NSCLC tumors from patients undergoing surgical resection in Japan, the United States or Australia having known *EGFR* or *KRAS* mutations and survival data were selected from a larger set of previously studied resected NSCLC [22], [23], [29]. In addition, we studied 45 DNA samples of resected lung adenocarcinomas from British Columbia Cancer Agency, Vancouver, Canada which had been studied by SNP arrays. An additional 19 resected NSCLC cases were obtained from Tartu University, Estonia. We also obtained 60 resected colorectal cancer samples from the University of Texas Southwestern Medical School Tissue Bank. Institutional Review Board permission and written informed consent were obtained from all patients at each collection site (the University of British Columbia - British Columbia Cancer Agency Research Ethics Board, University of Texas Southwestern Medical Center Institutional Review Board, University of Texas MD Anderson Cancer center Institutional Review Board, Ethics Review Committee on Human Research of the University of Tartu, Graduate School of Medicine and School of Medicine, Chiba University Ethical Committee, Graduate School of Medicine, Dentistry and Pharmaceutical Sciences, Okayama University Ethics Committee, and The Prince Charles Hospital Human Research Ethics Committee). The ethics committees of all institutes approved the individual study. Finally, in order to study tumor cell populations free of contaminating human stromal cells, we studied subrenal capsule xenograft samples in SCID mice directly established from primary human NSCLCs at British Colombia Cancer Center, Vancouver, Canada [30].

### DNA and RNA extraction

Genomic DNAs were isolated from cell lines, frozen tumors or paraffin embedded tumors (in 19 cases from Tartu University) by standard phenol-chloroform extraction [31] or by using DNeasy Tissue Kit (QIAGEN, Valencia, CA, USA). Total RNAs were extracted from cell lines using RNeasy Plus Mini Kit (QIAGEN). cDNA was prepared by reverse transcription of RNA using High-Capacity cDNA Reverse Transcription Kits (Applied Biosystems, Foster City, CA, USA) according to the manufacturer's protocol.

### Detection of gene mutations by direct sequencing

We determined the mutational status of *KRAS* (m*KRAS*), *BRAF* (m*BRAF*), *PIK3CA* (m*PIK3CA*) and *EGFR* (m*EGFR*) genes by direct sequencing as described previously [22], [23] and PCR conditions are provided in Table S3. Briefly, genomic DNA or cDNA was amplified by conventional PCR. All PCR products were incubated with exonuclease I and shrimp alkaline phosphatase (Amersham Bioscience Corp., Piscataway, NJ) and sequenced directly using the Applied Biosystems PRISM dye terminator cycle sequencing method (Perkin-Elmer Corp., Foster City, CA). All sequence variants were confirmed by sequencing the products of independent PCR reactions in both directions.

### Quantification of relative ratio between mutant and wild type alleles by direct sequencing

**Figure 2:**
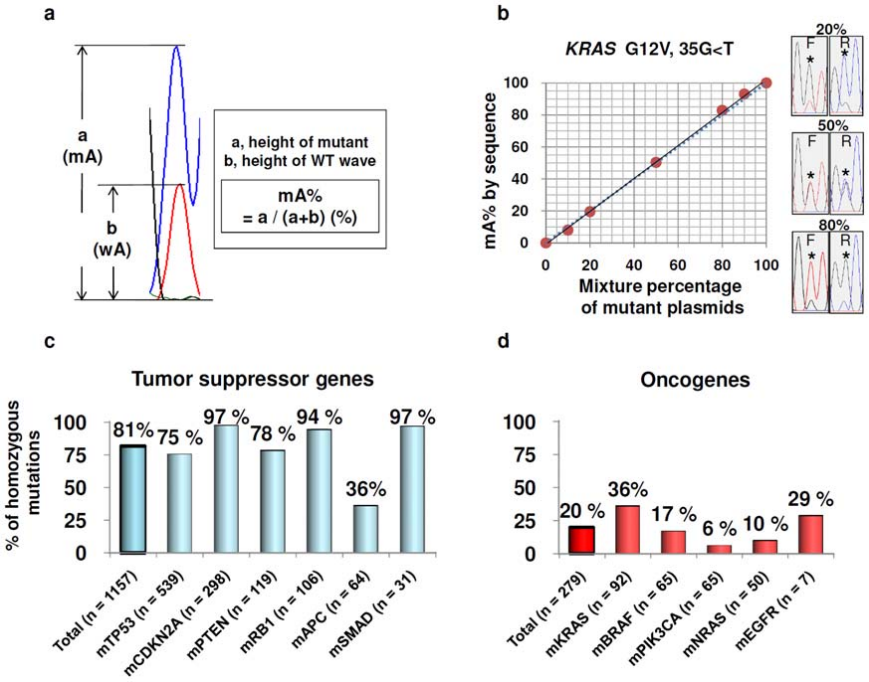
Homozygous mutations (complete MASI) of oncogenes are frequent. Quantitation of mutant allele (mA) by direct sequencing (a and b). wA, wild type allele; WT, wild type; mA%, proportion of mutant allele. a) Calculation method of mA% in point mutations by sequencing eletcropherogram is shown. b) An example of accuracy of mutant allelic quantitation (mA%) by measurement of sequencing electropherogram (*KRAS* mutation: G12V, 35G<T; results of forward reading is shown). We performed similar experiments for 14 kinds of mutations of *KRAS*, *BRAF*, *PIK3CA* or *EGFR* genes and confirmed the accuracy of mA% by measurement of sequencing electropherograms. F, forward sequencing; R, reverse sequencing c) and d) Frequency of homozygous mutations of 11 well-described tumor related genes in 833 cancer cell lines collected at Cancer Genome Project, Welcome Trust Sanger Institute (www.sanger.ac.uk/). As expected, homozygous mutations are frequent in six tumor suppressor genes (c). Those of five oncogenes are also relatively frequent (d). MASI, mutant allele specific imbalance; The prefix m- means mutant.

We quantified the relative ratios between mutant (mA) and wild type (wA) alleles by direct sequencing to determine the percent of the mutant allele (mA%) by three steps (Fig. 2a): 1) magnification of electropherogram on computer screen using Finch TV software (http://www.geospiza.com/finchtv.html) which can provide sharp wave lines without boldness after maximization, 2) pixel based wave peak heights measurement using a desktop ruler software, MB-Ruler (http://www.markus-bader.de/MB-Ruler/), and 3) calculation of mA%. For point mutations, we used the following formula: mA% = H_mut._/(H_mut_+H_wt_) (%), where H_mut_ is the minimum distance between midpoint of mutant wave line at peak and midpoint of baseline, and H_wt_ is the minimum distance between midpoint of wild type wave line at peak and midpoint of baseline. For deletion or insertion types of mutations, we used the average of mA% of the first five different waves from the beginning of mutations (Figure S1). We repeated the sequencing if the first sequencing eletropherogram demonsrated high background noise.

### Plasmids construction and plasmid mixture experiment

In order to validate mA% detected by direct sequencing, we constructed each mutant or wild type pCR2.1-TOPO plasmid from cell lines harboring 14 kinds of mutations using TOPO TA cloning kit (Invitrogen, Carlsbad, CA, USA) and QIAprep Miniprep Kit (QIAGEN). We mixed mutant plasmid with corresponding wild type plasmid at various ratios and amplified the mixed plasmid as a template of PCR using paired primer sets for mutational analyses. PCR products were directly sequenced and the mA% were determined by measurement of sequeincing electropherograms. Finally, we confirmed the linearity between the actual mixed proportion of mutant and wild type plasmids and mA% detected by direct sequencing (Fig. 2b).

### Quantification of relative ratio between mutant and wild type alleles by sub-cloning

PCR products were cloned into pCR2.1-TOPO vector using TOPO TA cloning kit (Invitrogen). About 20 clones (range15–25) were randomly selected for sequencing using either M13 forward primer or corresponding primers of each gene. mA% was calculated as the percentage of mutant clones in the total number cloned.

### Quantification of relative ratio between mutant and wild type alleles by restriction fragment length polymorphism (RFLP)

Genomic DNAs from mutant samples were amplified by PCR using corresponding primers which we have previously reported (Table S3) [3], [32], [33]. While mA of *EGFR* exon 19 deletion type mutations could be distinguished from wA based on 9 to 12 base pairs differences, overnight digestion of PCR products was needed for point mutations using appropriate enzymes which can specifically digest wild type sequences (Figures S2a and b). After 12.5% polyacrylamide gel electrophoresis, the gel was stained with ethidium bromide. Band intensity of the respective mA and wA was calculated using Kodak Image Station 2000RT and Kodak 1D Image Analysis Software (Kodak, Rochester, NY) and mA% was determined from these ratios. We also confirmed that multiple control samples (wild type) were completely digested in every assay (Figures S2a and b).

### Analyses of copy number by quantitative PCR assay

CNGs of *KRAS*, *EGFR*, *BRAF* and *PIK3CA* genes were determined by real-time quantitative PCR (qPCR) assay using Power SYBR® Green PCR Master Mix (Applied Biosystems) as previously reported (primer sequences are provided in Table S3) [22]. Briefly, we used *LINE-1* gene, which is the most abundant autonomous retrotransposon in the human consisting of 17% of the genome [34], as a reference gene for all copy number analyses. Gene dosage of each target and reference gene was calculated using the standard curve method. Relative copy number of each sample was determined by comparing the ratio of target gene to *LINE-1* in each sample with the ratio of these genes in normal human genomic DNA (EMD Biosciences, Darmstadt, Germany), made from a mixture of human blood cells from six to eight different donors, as a diploid control. Based on our previous study [6], we defined CNG in cell lines as values greater than four.

### Single nucleotide polymorphism (SNP) array and data processing

Samples were analyzed using the Genome-Wide Human SNP Array 6.0 platform (Affymetrix Inc., Santa Clara, CA) according to the manufacturer's directions. GeneChip Command Console Software (GCOS) was used to generate feature extracted intensity (.CEL) files which were subsequently processed using the Birdseed v2 algorithm in Genotyping Console 3.0.2 to create genotype (.chp) call files.

### Analysis of copy number and allelic imbalance by SNP array

Copy number and allele status were determined using Partek Genomics Suite (Partek Inc, St. Louis, MO). All CEL files were imported using the same default parameters. Copy number values were generated by normalizing each sample's probe set intensity to that of a reference. For tumors, paired references were used consisting of the normal lung tissue profile matching each patient. For lung cancer cell lines, an unpaired, pooled reference generated from the intensities of all 45 normal lung tissue profiles (those matching the tumors described above) was used. Regions of copy number gain and loss were then statistically detected using the Hidden Markov Model (HMM) based segmentation method of the software package with default parameters and the requirement of at least 50 contiguous probe sets.

Regions of allelic imbalance were determined using the allele specific copy number (AsCN) function of Partek. For paired analysis, only heterozygous SNPs in the reference (matched normal lung sample) were considered informative and the reference intensity for copy number creation was the allele intensity in the normal sample. In unpaired analysis, this reference intensity was taken as the average allele intensity of all reference (45 normal lung samples, see above) samples that were heterozygous for a given SNP. The ethnicity of all patients is listed in Table S2. Proportion scores for each SNP were then calculated and segmented in order to find regions of similar status and segments with a mean proportion score for all SNPs in the region >0.15 (as recommended by Partek) were considered imbalanced. Finally, adjacent regions meeting this threshold of imbalance were merged and the average proportion score calculated. The segment displaying the highest degree of imbalance across a chromosome arm (based on proportion score) is also listed for specific examples. All SNP data was visualized using SIGMA^2^ software (http://www.flintbox.com/technology.asp?page=3716) [35].

### mRNA expression of *KRAS*, *EGFR*, *BRAF* and *PIK3CA* genes by qPCR assay

mRNA expression of each mutant gene was evaluated by real-time qPCR of cDNA product. Primer sequencing and PCR conditions are provided in Table S3. As an internal control, we used glyceraldehyde 3-phosphate dehydrogenase (GAPDH) gene. After quantitation of each target and reference genes by the standard curve method, relative expression was calculated to compare the value of cell lines with the average value of HBEC 15 and 21 cell lines (for NSCLCs lines) or the value of human adult normal colon RNA (BioChain Institute, CA, USA) for CRC lines, respectively.

### Ras activity by ELISA

Ras activity was evaluated using Ras GTPase Chemi ELISA (Active Motif, CA) following the manufacturer's protocol. Briefly, cell lysates from cell lines were quantified using BSA Protein Assay Kit (Pierce, IL). Glutathione-S-transferase (GST) fused to ras-binding-domain (RBD) of Raf which can specifically bind only to activated Ras was coated onto glutathione-coated microplates by a one hour incubation. After washing, equal amounts of cell lysates (45 µg) were applied and incubated for one hour. A primary antibody which can detect H- and K-ras was added and incubated for one hour. An hour incubation with a second antibody conjugated to horseradish peroxidase (HRP) and developing chemiluminescent reagents were used to detect activated Ras binding to the plate. The luminescent intensity, which was inversely proportional to the amount of activated Ras, was read using FLUOstar OPTIMA (BMG LABTECH GmbH, Offenburg, Germany). Each cell line was tested in duplicate. All values presented are relative light units compared with mean value of two HBEC lines that was arbitrarily assigned a value of one.

### Estimation of tumor heterogeneity by SNP array

Tumor samples contain varying numbers of stromal and other non malignant cells that may affect estimates of tumor cell gene copy number and allelic imbalance. To estimate tumor DNA content for clinical samples, we used a method adapted from Weir et al [7]. Briefly, we determined the log_2_ ratios and LOH status for each informative SNP in the tumor samples using dChip software with default settings. Regions of hemizygous deletion (i.e. one copy loss in diploid cells) in each sample were determined by identifying SNPs that displayed copy number loss (tumor vs normal log_2_ ratio ≤−0.2) with concordant LOH. In order to identify the lost allele in these regions, we then calculated allele-specific intensity ratios using the aroma.affymetrix package in R [36]. Since the lost allele in these regions has zero copies, any signal would be attributed to contamination by normal cells (which have one copy of each allele). Thus, this lost allele ratio represents the percent of normal cells in the sample. For each sample, the median ratio of the lost allele was then calculated for individual chromosomes and the minimum of the medians was determined. This value was then subtracting from one to determine the percentage of tumor cells in each clinical sample.

### Statistical analyses

The differences of significance among categorized groups were compared using Chi-square or Fisher's exact tests as appropriate for univariate analyses. Univariate analyses of overall survival (OS) were performed using the Kaplan-Meier method with a log-rank test. All data were analyzed with GraphPad Prism 5 software (GraphPad Software, San Diego, CA). All statistical tests were two-sided and probability values <0.05 were defined as being statistically significant.

## Results

### Homozygous mutations (Complete MASI) of oncogenes are frequent in tumor cell lines

For the 11 genes queried in the Sanger database, we identified a total of 1436 mutations (1157 for tumor suppressor genes, 279 for oncogenes)(Table 1, Fig. 2c and 2d). As expected, homozygous mutations were frequent in six tumor suppressor genes (81%), with the exception of *APC*, while the five oncogenes also had a relatively high frequency of homozygous mutations (20%). However, the frequency of homozygous mutations varied - being frequent in *KRAS* or *EGFR* mutant lines but not with *PIK3CA* mutations. As shown below, the true incidence of MASI is higher, as the Sanger data base does not have quantitative copy number data for cell lines.

**Table 1:** Homozygous mutations of oncogenes are frequent in cancer cell lines.

	Sanger Institute	Our data
Genes	% of Homozygous mutations*	% of Homozygous mutations**
	(No. of mutant lines)	(No. of mutant lines)
Total	20 (279)	27 (75)
*KRAS*	36 (92)	38 (45)
*BRAF*	17 (65)	13 (8)
*PIK3CA*	6 (65)	0 (12)
*NRAS*	10 (50)	- (-)
*EGFR*	29 (7)	20 (10)

* , Zygosity status was determined by manual examination of sequencing electropherograms at Sanger institute; **, Homozygous mutations were defined as percent of mutant allele by direct sequencing greater than 90%.

We used the data from our cell lines to confirm these findings for four oncogenes (total of 75 mutations)(Table 1). We found a mean incidence of 27%, range 0% for *PIK3CA* to 38% for *KRAS*. The frequencies of homozygous mutations for *EGFR* (20%) and *BRAF* (13%) were intermediate. Thus our findings are similar and complementary to the information from the Sanger database.

### Determination of relative ratio between mutant and wild type alleles by direct sequencing

As described in the Methods Section, we determined the relative proportions of mutant and wild type alleles (mA%) by measurements of the direct sequencing eletropherograms. To validate this approach, we applied it to mixtures of varying percentages of wild type and mutant plasmids. The results of the sequencing method were highly concordant with the actual mixture percentage of mutant and wild type plasmids for all 14 mixture experiments for all four genes tested (R^2^ value≥0.95, Fig. 2b and Table S4). Furthermore, mA**%** of subcloning of 48 mutant lines (R^2^ value≥0.87) and RFLP analyses of 38 mutant lines (R^2^ value≥0.89) also showed good concordance with electropherogram measurements (Figure S2c), demonstrating the accuracy of latter assay. These results fully validate the sequencing eletropherogram measurement as an accurate method to determine mA%.

### Estimation of tumor DNA content in clinical samples

We estimated tumor DNA content (% tumor DNA) from the SNP array data as described in Methods for 45 lung adenocarcinomas. Two control NSCLC lines (100% tumor cells) had estimated values of 89% and 95% of % tumor DNA while the median value of the tumors was 57%, range 26 to 93%. For these 45 cases, we adjusted all copy number using the % tumor DNA.

### Mutations and CNGs of *KRAS*, *BRAF*, *PIK3CA*, and *EGFR* genes

**Figure 3:**
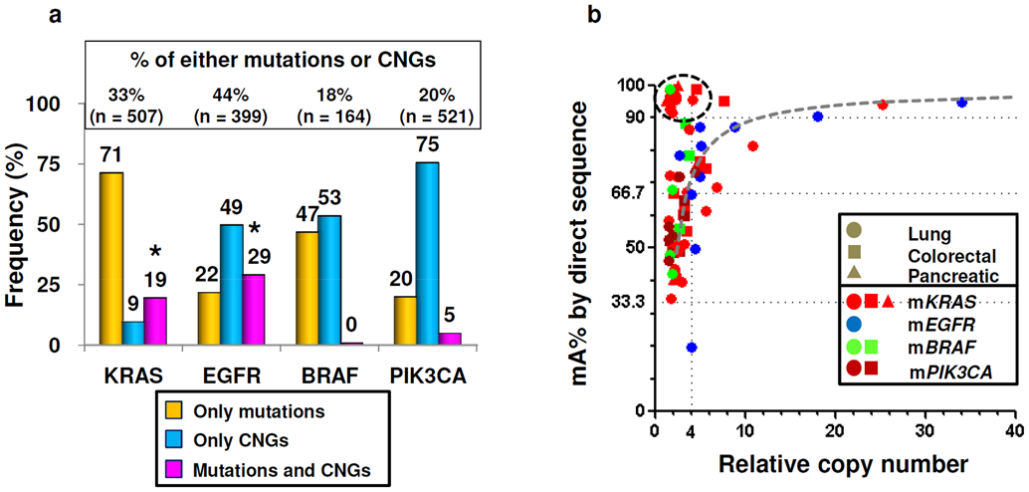
The association between mutations, copy number gain (CNG) and mutant allele specific imbalance (MASI) of *EGFR* pathway genes. a) The association between mutations and CNG of *EGFR* pathway genes in cell lines and tumors across organs. We combined the data of cell lines and tumors because of similarity of both data sets. Mutations are more frequent than CNG in *KRAS* gene while CNG are more frequent than mutations in other genes. CNG are significantly correlated with mutations in *KRAS* (*P*<0.0001) and *EGFR* (*P*<0.0001) genes (*). However, mutations or CNGs of *BRAF* and *PIK3CA* genes are usually exclusive and rarely present together. b) The association between percent of mutant allele (mA%) and copy number for 75 mutations in 68 mutant cell lines. Gray dotted line is the hypothetical curve of mutant allele specific amplification. There were 36 mutations with MASI (48%), 38 with balanced (51%) and one with reverse MASI (1%). Thirteen mutant cell lines including mutant *KRAS* (n = 12) and *BRAF* (n = 1) had uniparental disomy (complete MASI without CNG) and four lines (all mutant *KRAS*) had complete MASI with modest level of CNG (copy number<9, black dotted circle). The prefix m- means mutant; mA%, proportion of mutant allele.

Details of the gene mutations and CNGs in the cell lines (n = 114) and tumors (n = 521) are provided in Tables S1, S2 and S5. Without SNP array data, the presence of UPD in tumor samples could not be determined. Because the results of cell lines and tumors were similar, a combined summary is presented in Fig. 3a. All *KRAS*, *BRAF*, and *EGFR* mutations were mutually exclusive across different tumor types while some *PIK3CA* mutant cases also harbored one of the other three mutations (Tables S1 and S2) as described previously [22], [37], [38].

For the three genetic alterations (mutations, CNGs or both) each gene demonstrated a distinct pattern. Most of the alterations in *KRAS* were mutations, with occasional CNGs or both. For *EGFR*, CNGs were the most frequent alteration, although mutations or both changes were present in prominent subpopulations. For *BRAF*, mutations and CNGs showed nearly equal frequencies, while both changes were rare. For *PIK3CA*, CNGs without mutations were the most frequent change (Fig. 3a).

### The different patterns of MASI

The relationships between of mA**%** (as determined by electropherogram mesurement) and CNGs (as determined by qPCR) for the mutant genes in 68 mutant lines including seven lines with double mutations are shown in Fig. 3b. Three major patterns were observed: 1) Balanced, having a mA: wA ratio (mA/wA) of about 1 (range 0.5 to 2) without CNG (i.e. – MASI not present); 2) MASI with CNG, either complete [wA lost (mA/wA>9) or partial (mA/wA>2)]; and 3) MASI without CNG (uniparental disomy; UPD), due to complete loss of wA (mA/wA>9) and selective retention/duplication of mA, respectively (Fig. 1a and 3b). Cases with UPD or complete MASI with CNGs lie off the standard curve because they lack the wA (Fig. 3b). A fourth pattern, reverse MASI, defined as wild type allele specific imbalance (mA/wA<0.5) was present in only one line (1%) having a m*EGFR*.

### Gene specific analyses versus genome wide analyses

**Figure 4:**
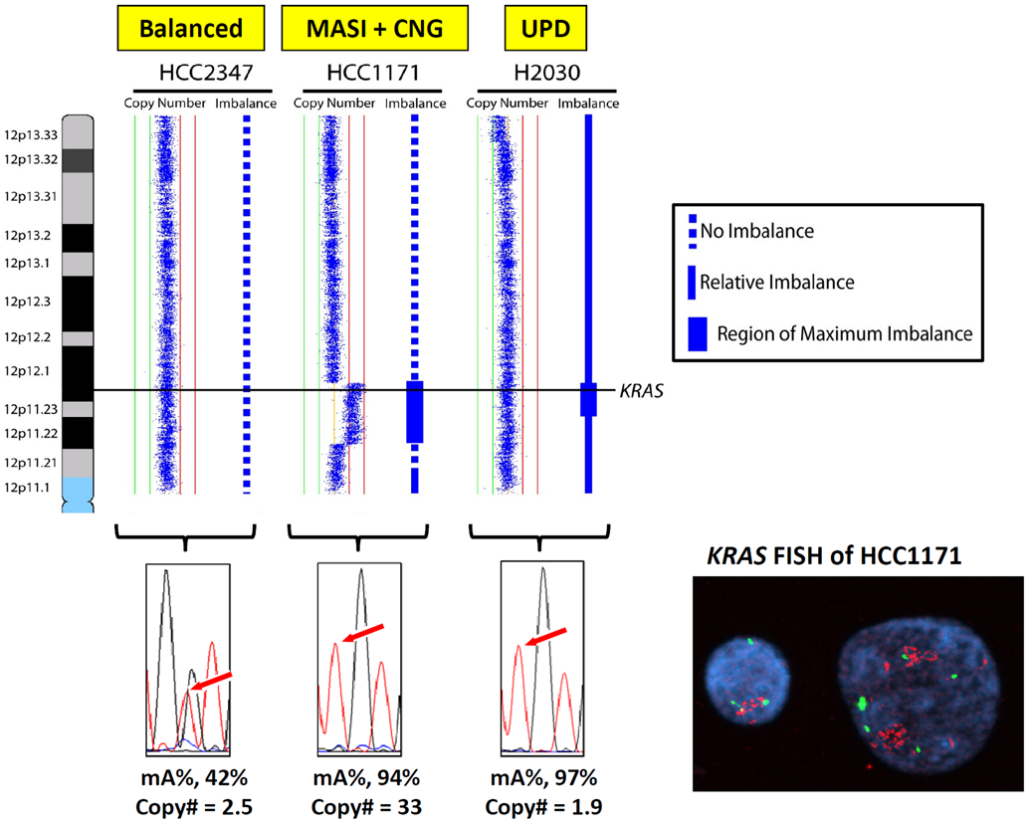
*KRAS* mutant allele specific imbalance (MASI) in lung cancer cell lines. (Left upper) Copy number and allelic imbalance status as determined by SNP 6.0 arrays are depicted for representative cell lines with balanced and MASI patterns of *KRAS* mutant/wild type allele ratios. For copy number, each blue dot represents an array element ordered by genomic position. Those shifted to the left of the middle line have decreased copy number whereas those shifted to the right have increased copy number. For allelic imbalance, dashed lines represent regions with no imbalance whereas solid lines represent those with imbalance. Thicker solid lines represent the region of maximum imbalance across the chromosome arm (see methods). The genomic location of *KRAS* is indicated by the horizontal black line. (Left lower) Electropherograms of direct DNA sequencing with mutant allele proportion (mA%, determined by electropherogram) and *KRAS* copy number (copy#, determined by quantitative PCR) are present in the same cell lines used for SNP arrays. (Right lower) *KRAS* FISH in HCC1171 was performed using purified DNA from BAC clone RP11-1119I8 encompassing the *KRAS* gene (red signal) and CEP12-SpectrumGreen (Abbott Molecular, IL) as an internal control. Means of *KRAS* copy number are 21.6±11.0 (standard deviation, SD) and those of CEP12 number are 3.7±1.2 (SD). Both SNP arrays and gene specific assays confirm that HCC2347 displays neutral *KRAS* copy number with no imbalance (mutant/wild type balanced) whereas HCC1171 and H2030 display imbalance (MASI) with copy number gain (CNG) or uniparental disomy (UPD), respectively.

We evaluated MASI status in seven m*KRAS* and two m*EGFR* lines using SNP arrays, and compared the results with MASI status determined by gene specific assays (mA% by direct sequencing and copy number by qPCR). Examples of these comparisons are shown in Fig. 4. Gene specific analyses defined the seven m*KRAS* lines as one balanced type, one having MASI with CNG and five having UPD. Of the m*EGFR* lines, one had MASI with CNG and one had MASI with borderline CNG. Of note, the results detected by SNP array were completely concordant with those of the gene specific assays.

### Individual oncogenes utilize different types of MASI

For our studies, determination of relative ratio between mA and wA (mA%) of tumor samples (in contrast to cell lines) requires SNP array analyses. As shown in Table S2b, we confirmed that there was good concordance between CNGs as estimated by SNP and qPCR methods.

For 45 adenocarcinomas having SNP array data, direct sequencing detected a high frequency of *KRAS* (n = 21, 47%) or *EGFR* (n = 14, 31%) mutations. We determined allelic imbalance (AI) and CNGs of *KRAS* and *EGFR* genes using SNP data. The percentage of tumor cell DNA in the samples was determined as described previously and we used appropriately adjusted copy numbers for further analyses. Because MASI frequencies in tumors (as determined by SNP assays) and cell lines (as determined by direct sequencing combined with qPCR) were similar (Table S6), we combined the data from 35 mutant tumors and 68 mutant cell lines.

**Figure 5:**
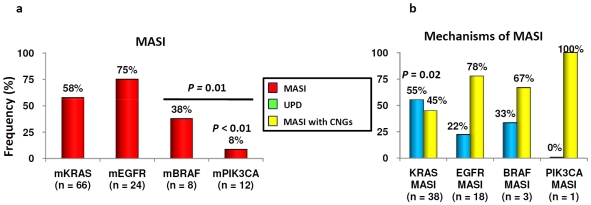
Different frequencies and mechanisms of MASI of *EGFR* pathway genes. MASI is equally frequent in mutant *KRAS* and *EGFR* genes than others and *PIK3CA* MASI is rare (a). *KRAS* MASI is caused almost equally by uniparental disomy or copy number gain (CNG) while *EGFR* MASI is mainly caused by CNG (b). The prefix m- means mutant. MASI, mutant allele specific imbalance.

As shown in Fig. 5a and Table 2, the frequencies for MASI (of all types) varied between individual oncogenes, being relatively high for *EGFR* (75%) and *KRAS* (58%) and lower for *BRAF* (38%) and *PIK3CA* (8%). The major type of MASI also showed gene variation (Fig. 5b and Table 2). For *KRAS*, UPD were more frequent than CNGs, while for *EGFR* the major type of MASI found in tumors and cell lines was CNGs, with UPD present in a minor subpopulation. For *BRAF* and *PIK3CA* the data were too scant to come to conclusions.

**Table 2:** Summary of allelic imbalance of *EGFR* pathway genes.

	Subsets	*KRAS*	*EGFR*	*BRAF*	*PIK3CA*
Mutant	Frequency of MASI	Frequent	Frequent	intermediate	Rare
	Mechanisms of MASI	UPD (+CNG)	CNG	CNG (+UPD)	CNG
Wild type	Frequency of AI in WT	Equally frequent as MASI	Rare	-	-
	Mechanisms of AI	UPD	Rare (CNG)	-	-

AI, allelic imbalance; MASI, mutant allele specific imbalance; WT, wild type; UPD, uniparental disomy; CNG, copy number gain.

### Allelic imbalance can be equally observed in wild type *KRAS*

We determined AI in both wild type and mutant case for *KRAS* and *EGFR* genes among the 45 lung adenocarcinomas with SNP data. For all 45 cases, AI was frequent in *KRAS* (n = 28, 56%) and *EGFR* (n = 18, 40%)(Table S2b). However, *EGFR* AI was significantly more frequent in m*EGFR* (71%) than wild type *EGFR* cases (29%, *P* = 0.008). By contrast, AI of *KRAS* was equally observed in m*KRAS* (62%) and wild type *KRAS* (63%, Table 2). While *EGFR* AIs in wild type *EGFR* cases were equally caused by CNG (50%) and UPD (50%), all *KRAS* AIs in wild type *KRAS* cases were caused by UPD.

### Double mutations occur on the same chromosome (*cis* mutations)

Second site (double) mutations in the same gene (two examples each for *EGFR* and *PIK3CA*) were present in four cell lines (Table S1). For all four cell lines they showed very similar mA% for both sites (less than 3.5% difference)(Table S1), even though two mutations of *EGFR* were detected by independent PCR reactions. These findings suggested that in all four cases both mutations occurred on the same parental chromosome and were in *cis* with each other. For *EGFR* mutant cases, a common activating mutation was associated with a second resistance associated mutation (T790M) and these two mutations have been described as usually or always being in *cis* [39], [40].

### MASI is present in xenografts

Subrenal capsule mice xenografts were directly established from primary human NSCLCs. These samples have the following advantage: 1) less manipulation than cell lines (close to clinical samples), and 2) lack of human normal stromal contamination [30]. We identified two *KRAS* or two *EGFR* mutations by cDNA sequencing using primer sets specific for the human gene in four of 27 subrenal xenograft samples (Figure S3). We confirmed the human specificity of our primers by lack of an amplicon using cDNA from healthy non-manipulated mouse liver as template (data not shown). Of note, three out of four mutant samples (two *KRAS* and one *EGFR* mutations) showed over 90% of mA%.

### The ratio of mutant: wild type allele is maintained after transcription

**Figure 6:**
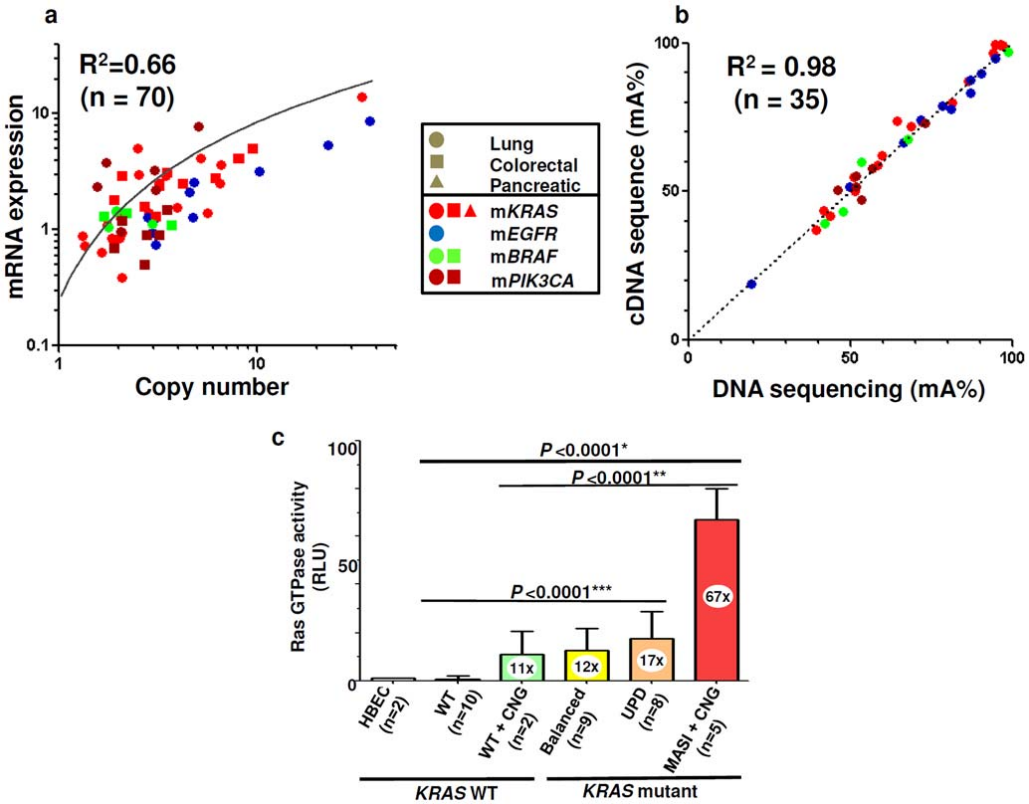
Biological role of mutant allele specific imbalance (MASI). Gene dosage is highly associated with mRNA expression level (a). Proportion of mutant allele (mA%) determined by DNA sequencing electropherogram is significantly consistent with mA% by cDNA sequencing using different sets of primers (b). c) *KRAS* alterations are related to ras GTPase activity. *KRAS* mutations or copy number gains (CNGs) alone are related to high ras GTPase activity and the two molecular changes are synergistic. The prefix m- means mutant. HBEC, human bronchial epithelial cell; WT, wild type; UPD, uniparental disomy; *, *KRAS* mutation with CNG versus Others; **, *KRAS* mutation with CNG versus either *KRAS* mutation or CNG; ***, either *KRAS* mutation or CNG versus WT.

To investigate whether CNGs were reflected in increased transcriptional activity, we compared mRNA expression with copy number for 70 mutant cell lines (with or without MASI). As shown in Fig. 6a, there was good concordance between the results of the two techniques, indicating that increased copy number was accompanied by increased transcription.

We then investigated whether the increased mA% of MASI lines were maintained after transcription. Using a subset of 35 mutant cell lines (with or without MASI), we compared the mA% of genomic DNA with the values from cDNA (Fig. 6b). There was almost perfect concordance between the values, indicating that the ratios of mutant:wild type alleles in genomic DNA were faithfully maintained after transcription.

### Ras GTPase activity and *KRAS* MASI

We evaluated ras GTPase activity by ELISA for 36 cell lines including 26 lung, five colorectal, three pancreatic cancer lines and two HBEC lines (Figure S4). The linearity of the standard curve made by five different points was confirmed (R^2^ = 0.97, data not shown). HBEC cultures and wild type tumor cell lines had comparably low levels of activity (Fig. 6c). Both lines with *KRAS* CNGs (without mutation) and those with balanced mutations (without CNGs) had significant 11–12 fold increases in GTPase activity. Cell lines having UPD (without CNGs) had a modest (approximately 50%) increase compared to the balanced mutant lines, although this increase was not significant. However mutant lines having MASI with CNGs had a significantly increased mean activity when compared to the other mutant groups.

### *EGFR* MASI and in vitro sensitivity to gefitinib

We have previously reported the gefitinib sensitivity of NSCLC lines [6]. Seven of the 10 *EGFR* mutant lines were sensitive at a clinically achievable concentration (<1 µM). We correlated these data with the presence or absence of MASI (Table S7). While six out of seven sensitive cell lines (86%) harbored *EGFR* MASI, we could not find a convincing relationship between gefitinib sensitivity and *EGFR* MASI.

### *KRAS* mutations and copy number gains in lung adenocarcinomas

We determined the mutational status and copy numbers of *KRAS* gene for 288 lung adenocarcinoma tumors including Non-Asian (n = 127) and Asian (n = 161) populations obtained from five different institutions and correlated the data with clinical and other findings (Table 3). *EGFR* mutational status was available for 269 out of 288 cases [22], [23], [29]. We identified 57 *KRAS* mutations (20%) and 29 *KRAS* CNGs (10%). As demonstrated previously in Fig. 3a (for both cell lines and tumors), in this subset of tumors *KRAS* CNGs were more frequent in m*KRAS* than in wild type tumors. Because *KRAS* CNGs were closely associated with m*KRAS*, *KRAS* CNGs demonstrated similar associations as have been previously described for m*KRAS* (non-Asian ethnicity, smoking history, and mutual exclusivity with *EGFR* mutations). Gender differences were not significant for either mutations or CNGs.

**Table 3:** The association between *KRAS* alterations and clinical and other genetic factors in 288 lung adenocarcinomas.

*KRAS* mut	*P*	Subsets (n)	%	*KRAS* CNG	*P*	Subsets	%	Mut or CNG	*P*	Subsets	%	Mut and CNG	*P*	Subsets	%
All	-	- (288)	19.8	All	-	-	10.1	All	-	-	26.1	All	-	-	3.8
Gender	NS	Male (161)	21.7	Gender	0.08	Male	13	Gender	NS	Male	29.2	Gender	NS	Male	5.6
		Female (127)	17.3			Female	6.3			Female	22			Female	1.6
Smoking*	0.0018	Never (101)	9.9	Smoking*	0.013	Never	4	Smoking*	0.0001	Never	12.9	Smoking*	NS	Never	1
		Ever (184)	25.5			Ever	13			Ever	33.2			Ever	5.4
Ethnicity	0.0006	Non-Asian (127)	29.1	Ethnicity	NS	Non-Asian	13.4	Ethnicity	<0.0001	Non-Asian	37.8	Ethnicity	NS	Non-Asian	4.7
		Asian (161)	12.4			Asian	7.5			Asian	16.8			Asian	3.1
*EGFR* mut**	<0.0001	Mutant (65)	0	*EGFR* mut**	0.008	Mutant	1.5	*EGFR* mut**	<0.0001	Mutant	1.5	*EGFR* mut**	NS	Mutant	0
		WT (204)	25			WT	12.6			WT	32.4			WT	2.9

Mut, mutation; WT, wild type; CNG, copy number gain; NS, not significant; *, Smoking status was not available in three cases; **, Nineteen cases were not determined mutational status and copy number of *EGFR* gene.

**Figure 7:**
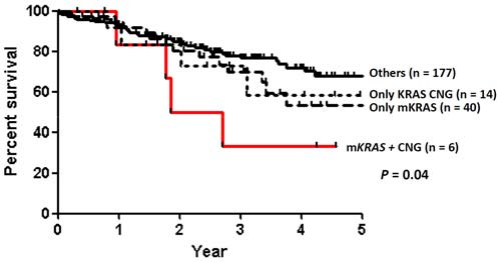
The effect of *KRAS* mutations and copy number gain (CNG) on clinical outcome in 237 lung adenocarcinomas. The effect of 1) *KRAS* mutations (without CNG), 2) *KRAS* CNG (without mutations), 3) both *KRAS* mutations and CNG, and 4) others (without *KRAS* mutations and CNGs) on clinical outcome is shown. Tumors having both alterations indicate worse prognosis with borderline significance than all others (*P* = 0.04). The prefix m- means mutant.

We then evaluated the effect of *KRAS* alterations on clinical outcome of 237 resected lung adenocarcinoma tumors which were limited to stage I–III cases with survival data. Patients with m*KRAS* tumors (*P* = 0.2) or *KRAS* CNGs (*P* = 0.1) alone had a trend to be associated with poor prognosis. Tumors having both alterations, while present in a small subpopulation (n = 6), had worse prognosis of borderline significance (*P* = 0.04, Fig. 7).

We also identified 105 *EGFR* CNG in same subset of 269 lung adenocarcinomas. *EGFR* CNGs were significantly more frequent in never smokers, Asian ethnicity, were mutually exclusive with *KRAS* mutations and occurred more frequently with *EGFR* mutations than in wild type cases as previously described [26]. We were unable to investigate the effects of *EGFR* mutations and CNGs on survival as data on TKI therapy was incomplete.

## Discussion

Our earlier observations regarding homozygous mutations and MASI led us to question the commonly held belief that tumorigenesis requires biallelic inactivation for tumor suppressor genes while the potent effects of dominant oncogenes preclude the necessity of loss of the wild type allele product. We examined a public database of mutations (Sanger Institute). We found, as expected, that most inactivating mutations of tumor suppressor genes were frequently accompanied by loss of the wild type allele. However, our earlier observations on homozygosity of oncogenes were confirmed by the finding that 20% of five activating oncogene mutations were homozygous in cell lines derived from multiple tumor types. As discussed below, the true incidence of MASI is considerably higher as quatitative copy number data are missing in the Sanger database. Thus MASI, while a long observed and expected phenomenon for tumor suppressor genes, is also present in an important subset of cells harboring mutant oncogenes. Other published evidence supports this concept [41].

Detection of MASI of an oncogene requires three basic determinations: 1) detection of an oncogenic mutation; 2) copy number enumeration of the mutant gene in the tumor cells and 3) determination of the relative ratio of the mutant:wild type allele (mA%). Standard and widely accepted methods for the first two determinations exist including direct sequencing for mutations, and qPCR, FISH, aCGH or SNP analyses for CNGs [6]. For cell lines (consisting of pure tumor cell populations) mA% can be determined by subcloning or by the presence of homozygosisty of the mutant allele. In order to avoid laborious and time intensive subcloning, we determined that mA% could be accurately estimated by measurements of the relative peak heights present on the electropherograms of routine sequencing for mutation detection. While mA% could be determined accurately in cell lines by these simple techniques, tumor samples present a much greater problem because of contamination with highly variable percentages of non-malignant cells. Reports of molecular studies often provide estimates of the percentage of tumor cells by histologic examination, but these are usually performed rapidly and are relatively inaccurate. In addition, because of the frequent presence of tumor cell polyploidy, most genetic analyses require determination of the percentage of tumor DNA in the examined sample, rather than the percentage of tumor cells. For our studies, we used SNP array data for determinations of tumor cell DNA percentages. While this approach has been used by others [7], we refined the methodology. We found a mean value of 57% tumor DNA in the samples having SNP data, with a wide range of values. We arbitrarily used a slightly more conservative estimate for tumor cell DNA of 50% for the tumor samples lacking SNP data. While we used such estimates for copy number determinations in tumors, recognition of tumor homozygosity, including UPD, was limited to the tumor subsets with SNP data.

Four types of inter-relationships between mA and wA were found: a) balanced type, with mutant:wild type allele ratio of approximately one (MASI not present); b) MASI (either partial or complete) with CNG; c) uniparental disomy (complete MASI without CNG); and d) reverse MASI (wild type allele increased relative to mutant allele). For 75 mutations (in four genes) present in 68 cell lines the overall incidence of MASI was 48%, while only a single example of reverse MASI was identified (p<0.0001). Thus allelic imbalance almost invariably targets the mutant allele. Our previous observations regarding allelic imbalance (obtained by a variety of techniques including subcloning) are consistent with our present findings [3], [6]. While MASI was convincingly demonstrated in cell lines the true incidence in tumors could only be determined with accuracy for the subset of lung tumors having SNP array data and mutational status of the *KRAS* and *EGFR* genes. The incidences of MASI in lung cancer cell lines and tumors for these two genes were not significantly different. However, the incidences of MASI for individual oncogenes showed differences, with high frequencies for *EGFR* and *KRAS*, intermediate for *BRAF* and low for *PIK3CA*. These differences may reflect variations in the oncogenic potential of the individual gene mutations. The frequencies of the two major forms of MASI also demonstrated individual gene differences. For *EGFR* and *BRAF*, the most frequent type was MASI with CNGs, while for *KRAS*, the frequencies of MASI with CNGs and UPD were similar.

While mutations of the *KRAS* and *EGFR* genes and CNGs of the *EGFR* gene are well described [6], [19], [25], [26], [29], the literature regarding *KRAS* CNGs in human tumors is sparse [7], [42]. While less common than mutations in the present study, *KRAS* CNGs were relatively frequent. Of interest, *KRAS* CNGs showed the same clinico-pathological associations as those previously described for *KRAS* mutations – relationship to smoking status, non-Asian ethnicity and mutual exclusivity with *EGFR* mutations [29].

While inherited UPD is associated with developmental disorders. the role of acquired UPD in cancer development is poorly understood [12]. Although UPD has been reported to be related to inactivation of tumor suppressor genes, its presence with activating oncogenic mutations has rarely been described in tumors. To date, UPD has been mainly reported in hematopoietic malignancies for a few oncogenes such as *JAK2* [14]. Its incidence and role in solid tumors is largely unknown, although, as previously pointed out, this reflects the limits of our prior technology [12]. As discussed previously, homozygosity of tumor oncogenes in cancer cell lines is frequent, although the available information did not permit the distinction between MASI with CNGs or UPD as the mechanism. Using gene-specific and genome-wide approaches we found that UPD was frequent for three *EGFR* pathway genes, especially for *KRAS* gene (data for *PIK3CA* mutations were too sparse for evaluation). Relatively little data exists in the literature for *KRAS* CNGs in human tumors. Furthermore, *KRAS* homozygosity was observed independent of mutational status as previously described [43]. The wild type allele of *KRAS* can also inhibit lung carcinogenesis in mice [44], providing a possible explanation for the frequent finding of UPD with mutant and wild type oncogenes.

MASI has apparent biological and clinical significance. MASI at the genomic level was precisely maintained after transcription. While mutations, CNGs and allelic imbalance of mA and wA may all contribute to tumorigenesis, combinations of the three events may be more effective than any single event. Evidence for this concept was provided by our finding that the combination of mutation and CNGs acted synergistically to enhance ras GTPase activity. A recent report found that all *KRAS* mutations did not exert an equal effect on tumor cells [42]. Cancer cell lines harboring *KRAS* mutations could be broadly divided into *KRAS*-dependent and *KRAS*-independent groups. The vast majority of *KRAS*-dependent lines exhibited focal *KRAS* CNGs, in contrast to *KRAS*-independent lines. This study provides further evidence that the combination of *KRAS* mutations and CNGs act synergistically. Our previous findings that *EGFR* mutations were associated with tumor initiation while *EGFR* CNG might be more regarded as a tumor progression event, provide further evidence of their co-operative role in tumorgenesis [45]. Understanding the mechanism of MASI could elucidate new understandings of tumor biology and may contribute to the development of rational targeted therapies.

MASI in its various forms is frequently present in mutant *EGFR* and *KRAS* tumor cells, and is associated with increased mutant allele transcription and gene activity. The frequent finding of mutations, copy number gains and MASI occurring together in tumor cells indicates that these three genetic alterations, acting together, may have a greater role in the development or maintenance of the malignant phenotype than any individual alteration.

## Supporting Information

Table S1 a) Mutant cell lines of KRAS, EGFR, BRAF, and/or PIK3CA genes (n = 68) mA%, mutant allele proportion (%); *, cell line with both KRAS and PIK3CA mutations; **, cell line with both BRAF and PIK3CA mutations; ***, blanked values are mA% of second mutations of same gene (D549N for PIK3CA and T790M for EGFR)(For EGFR DNA sequence, we performed independent PCR reaction to evaluate mA% of primary and second mutations). b) Wild type cell lines of KRAS, EGFR, BRAF, and PIK3CA genes (n = 46)
(0.04 MB XLS)

Table S2 a) Summary of 288 lung adenocarcinomas from five institutes b) Summary of 45 lung adenocarcinomas with SNP array data c) The association between KRAS and EGFR alterations and clinicopathological factors in 45 lung adenocarcinomas with SNP *, P value was calculated between Gain and Neutral; **, P value was calculated between Never smoker and Ever smoker. d) Summary of 60 colorectal cancer tumors
(0.17 MB XLS)

Table S3 a) Primer sequences for DNA sequencing b) Primer sequences for cDNA sequencing *, These primers were also used to detect KRAS or EGFR mutations in subrenal capsule mice xenografts of primary human NSCLCs because these primers are specific for human origin and no PCR product are amplified from mouse cDNA as PCR template. c) Primer sequences for restriction fragment length polymorphism *, The substitution of third letter in KRAS codon 61 (limited to CAT or CAC mutation) can change representative amino acid (Gltamine to Histysine). d) Primer sequences for copy number analyses by quantitative PCR (qPCR) assay e) Relative mRNA expression analyses by qPCR
(0.03 MB XLS)

Table S4 The accuracy of proportion of mutant allele (mA%) of direct sequencing was evaluated by 14 kinds of plasmids mixture experiment. We mixed mutant plasmid with corresponding wild type plasmid at various ratios (5 to 7 points) and amplified the mixed plasmid as a template of PCR. PCR products were directly sequenced and the mA% were determined by measurement of sequeincing electropherograms. Finally, we confirmed the linearity between the actual mixed proportion of mutant and wild type plasmids and mA% detected by direct sequencing. The results of the sequencing method were highly concordant with the actual mixture percentage of mutant and wild type plasmids in all 24 trend lines for four genes tested (R2 value>0.95).
(0.02 MB XLS)

Table S5 CNG, copy number gain; Both, cases with both mutations and CNGs; NS, not significant (P>0.1); *, 314 tumors were analyzed because of lack of mutational and copy number data of EGFR gene in 19 Estonia cases; **, data were combined current study and our previous studies - Yamamoto et al (Cancer Res 68: 6913–6921) and Gandhi et al (PLoS ONE 4: e4576).
(0.02 MB XLS)

Table S6 CRC, colorectal cancer; PAC, pancreatic cancer; MASI, mutant allele specific imbalance; UPD, uniparental disomy; CNG, copy number gain; *, limited to 45 lung adenocarcinomas with SNP data; **, because SNP array can not distinguish between MASI and reverse MASI and because incidence of reverse MASI in cell lines is low, we defined tumors harboring allelic imbalance with CNG as MASI with CNG.
(0.02 MB XLS)

Table S7 All other 35 cell lines tested (except for 3 EGFR or HER2 copy number gain cell lines) were resistant for gefitinib (IC50>10 mM)(Gandhi et al: PLoS ONE 4: e4576)
(0.03 MB XLS)

Figure S1 Calculation method of mutant allele proportion (mA%) for deletion (or insertion) type of mutations is shown. The average of mA% of the first five different waves from the beginning of mutations is calculated.
(0.42 MB PPT)

Figure S2 We performed restriction fragment length polymorphism (RFLP) method to quantify mutant allele (Figures S2a and b). Examples for two types of mutations (KRAS codon 12 mutations and EGFR exon 19 deletion type mutations) are shown. Percent of mutant allele (%mA) detected by measurement of sequencing electropherogram has good concordance with %mA detected by subclonig and RFLP methods (Figure S2c).
(0.59 MB PPT)

Figure S3 Mutant allele specific imbalance (MASI) can be observed in mice xenograft samples. Complete MASI is present in xenogragts established from patients with stage Ib to IIIa.
(0.16 MB PPT)

Figure S4 Ras GTPase activity in 36 cell lines is shown. MASI, mutant allele specific imbalance; WT, wild type; CNG, copy number gain; HBEC, human bronchial epithelial cell; The prefix m- means mutant.
(0.18 MB PPT)
