# Supplementary material for: Oncogene Mutations, Copy Number Gains and Mutant Allele Specific Imbalance (MASI) Frequently Occur Together in Tumor Cells
Source: PLoS One. 2009 Oct 14;4(10):e7464. doi: 10.1371/journal.pone.0007464 (PMC2757721; doi:10.1371/journal.pone.0007464)
Supplement: Figure S1 — Calculation method of mutant allele proportion (mA%) for deletion (or insertion) type of mutations is shown. The average of mA% of the first five different waves from the beginning of mutations is calculated. (0.42 MB PPT) [file pone.0007464.s008.ppt]

## Slide 1
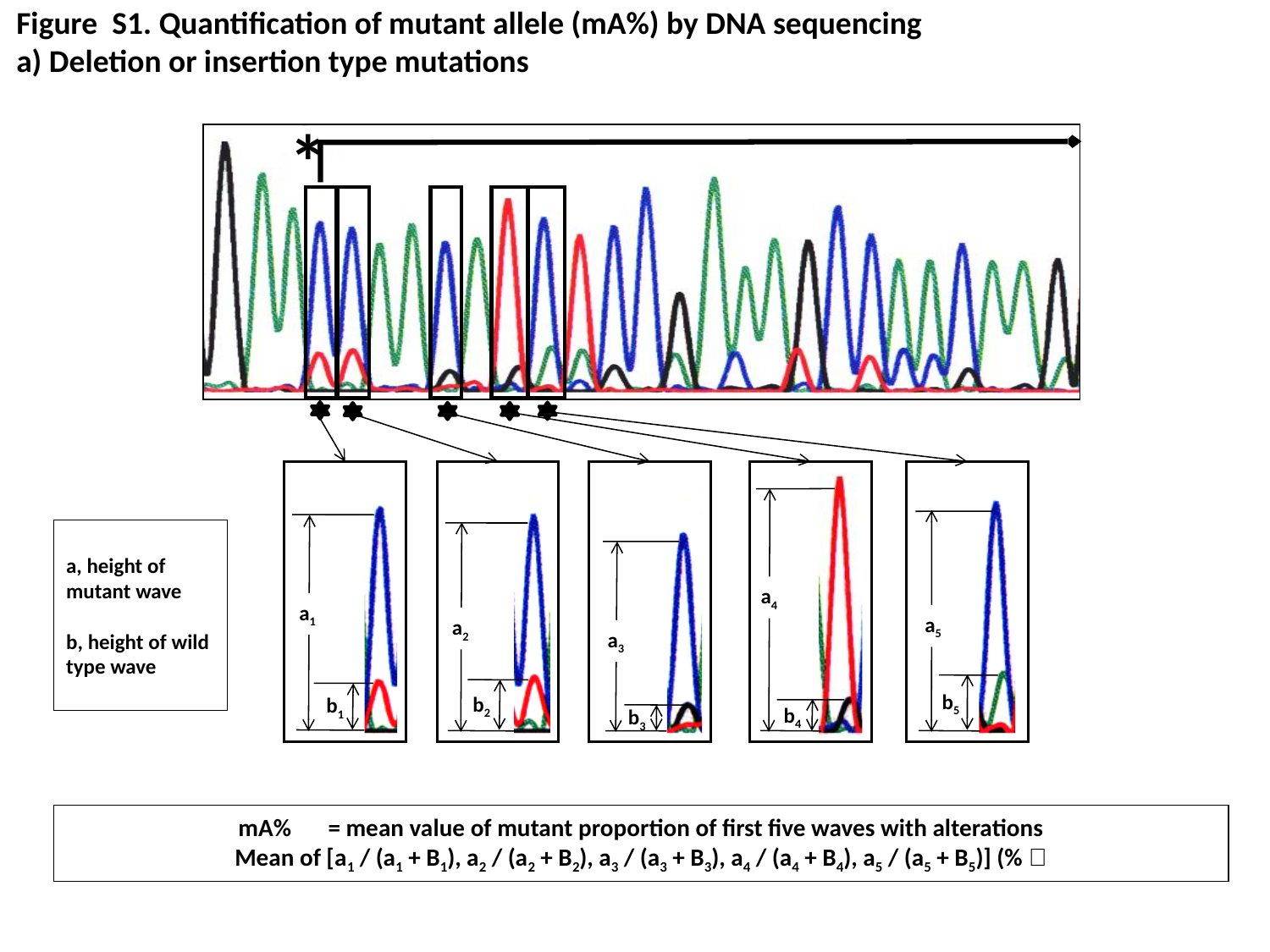

Figure S1. Quantification of mutant allele (mA%) by DNA sequencing
a) Deletion or insertion type mutations
*
a1
b1
a2
b2
a3
b3
a4
b4
a5
b5
a, height of mutant wave
b, height of wild type wave
mA%　= mean value of mutant proportion of first five waves with alterations
Mean of [a1 / (a1 + B1), a2 / (a2 + B2), a3 / (a3 + B3), a4 / (a4 + B4), a5 / (a5 + B5)] (%）
