# Supplementary figures and images for: Oncogene Mutations, Copy Number Gains and Mutant Allele Specific Imbalance (MASI) Frequently Occur Together in Tumor Cells
Source: PLoS One. 2009 Oct 14;4(10):e7464. doi: 10.1371/journal.pone.0007464 (PMC2757721; doi:10.1371/journal.pone.0007464)

## Slide 1
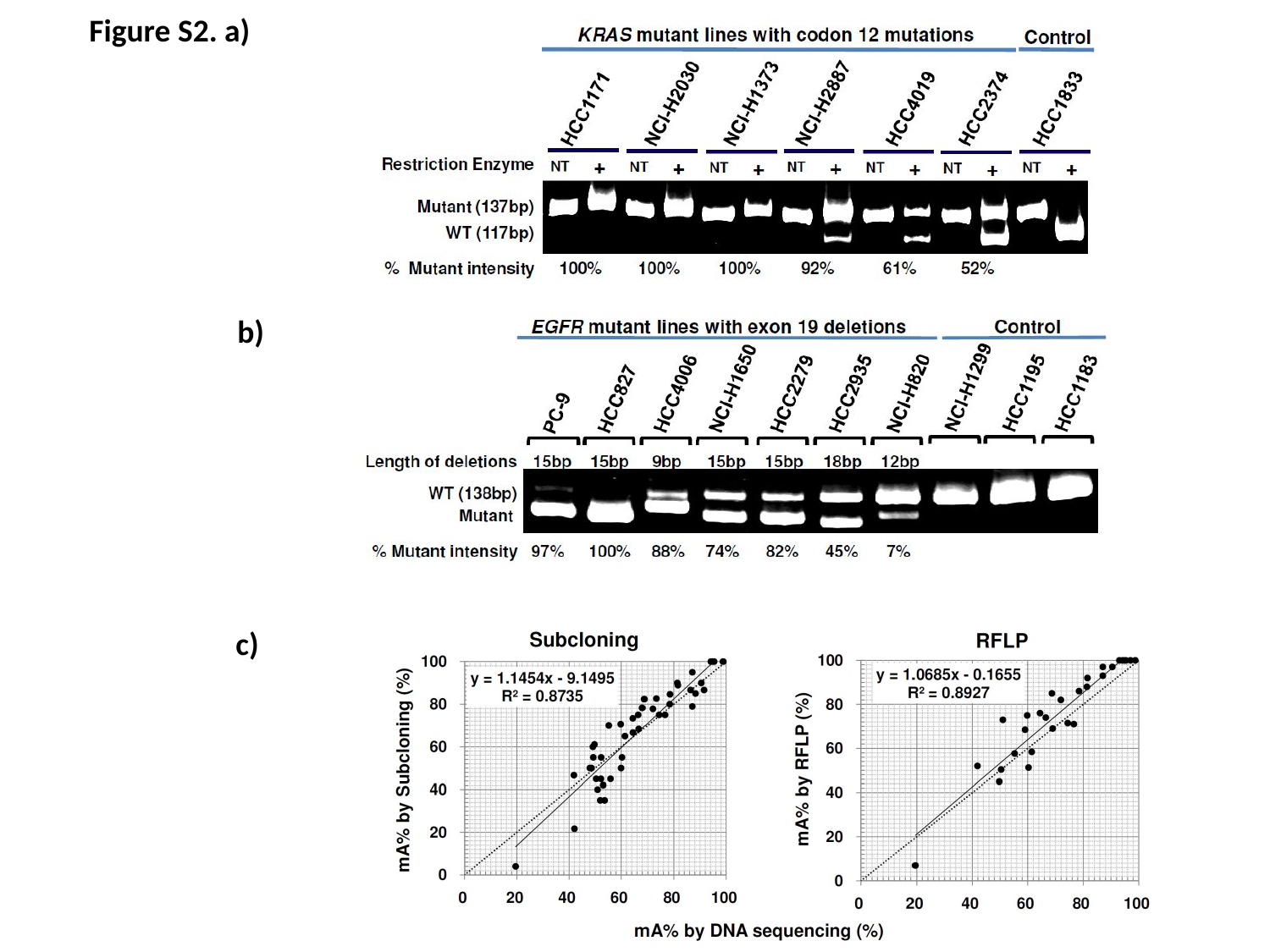

Figure S2. a)
b)
c)

Supplement: Figure S2 — We performed restriction fragment length polymorphism (RFLP) method to quantify mutant allele (Figures S2a and b). Examples for two types of mutations (KRAS codon 12 mutations and EGFR exon 19 deletion type mutations) are shown. Percent of mutant allele (%mA) detected by measurement of sequencing electropherogram has good concordance with %mA detected by subclonig and RFLP methods (Figure S2c). (0.59 MB PPT) [file pone.0007464.s009.ppt]
