# Supplementary material for: Oncogene Mutations, Copy Number Gains and Mutant Allele Specific Imbalance (MASI) Frequently Occur Together in Tumor Cells
Source: PLoS One. 2009 Oct 14;4(10):e7464. doi: 10.1371/journal.pone.0007464 (PMC2757721; doi:10.1371/journal.pone.0007464)
Supplement: Figure S3 — Mutant allele specific imbalance (MASI) can be observed in mice xenograft samples. Complete MASI is present in xenogragts established from patients with stage Ib to IIIa. (0.16 MB PPT) [file pone.0007464.s010.ppt]

## Slide 1
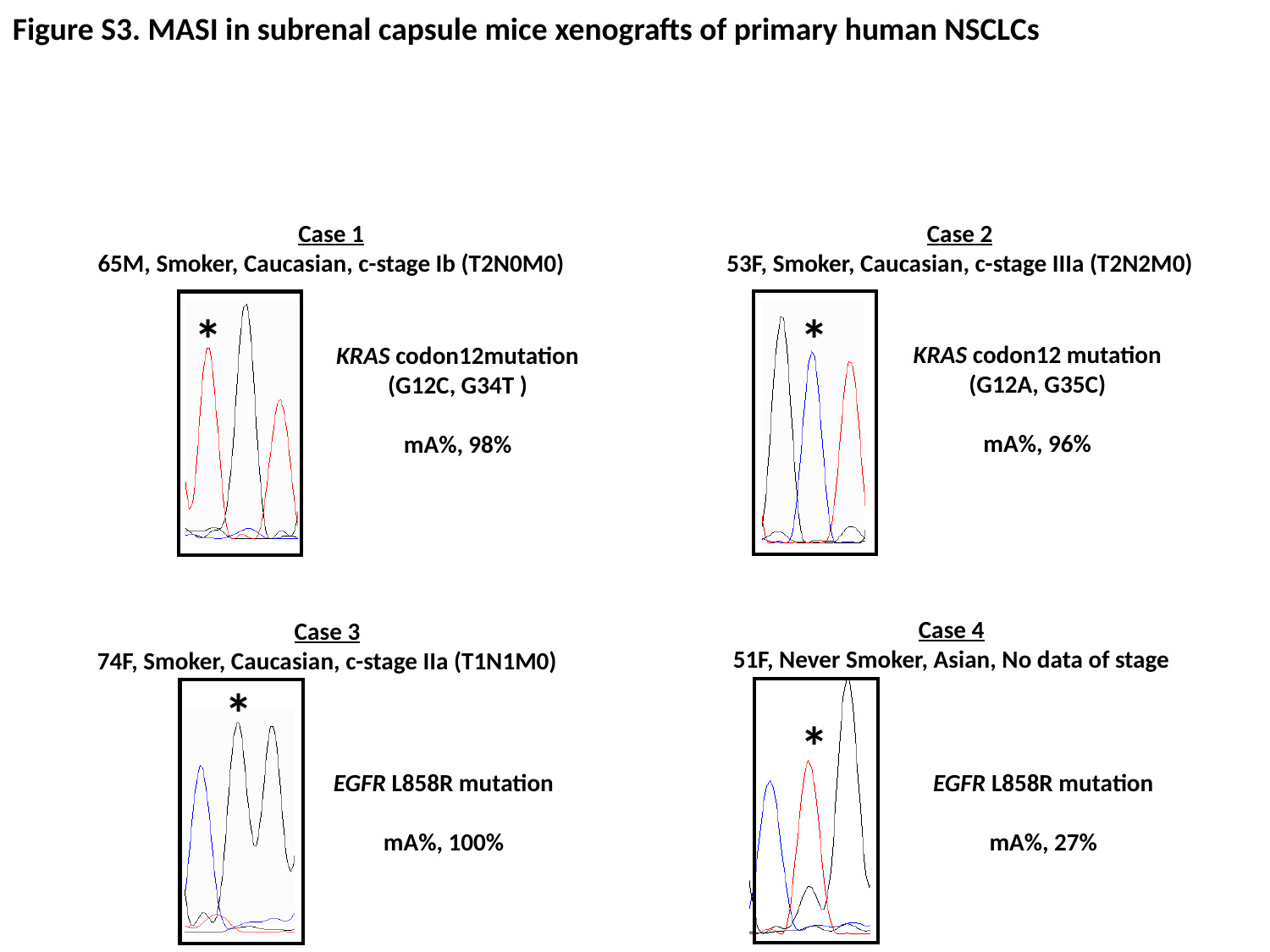

Figure S3. MASI in subrenal capsule mice xenografts of primary human NSCLCs
Case 1
65M, Smoker, Caucasian, c-stage Ib (T2N0M0)
Case 2
53F, Smoker, Caucasian, c-stage IIIa (T2N2M0)
*
*
KRAS codon12 mutation
(G12A, G35C)
mA%, 96%
KRAS codon12mutation
(G12C, G34T )
mA%, 98%
Case 4
51F, Never Smoker, Asian, No data of stage
Case 3
74F, Smoker, Caucasian, c-stage IIa (T1N1M0)
*
*
EGFR L858R mutation
mA%, 100%
EGFR L858R mutation
mA%, 27%
