# Supplementary material for: Oncogene Mutations, Copy Number Gains and Mutant Allele Specific Imbalance (MASI) Frequently Occur Together in Tumor Cells
Source: PLoS One. 2009 Oct 14;4(10):e7464. doi: 10.1371/journal.pone.0007464 (PMC2757721; doi:10.1371/journal.pone.0007464)
Supplement: Figure S4 — Ras GTPase activity in 36 cell lines is shown. MASI, mutant allele specific imbalance; WT, wild type; CNG, copy number gain; HBEC, human bronchial epithelial cell; The prefix m- means mutant. (0.18 MB PPT) [file pone.0007464.s011.ppt]

## Slide 1
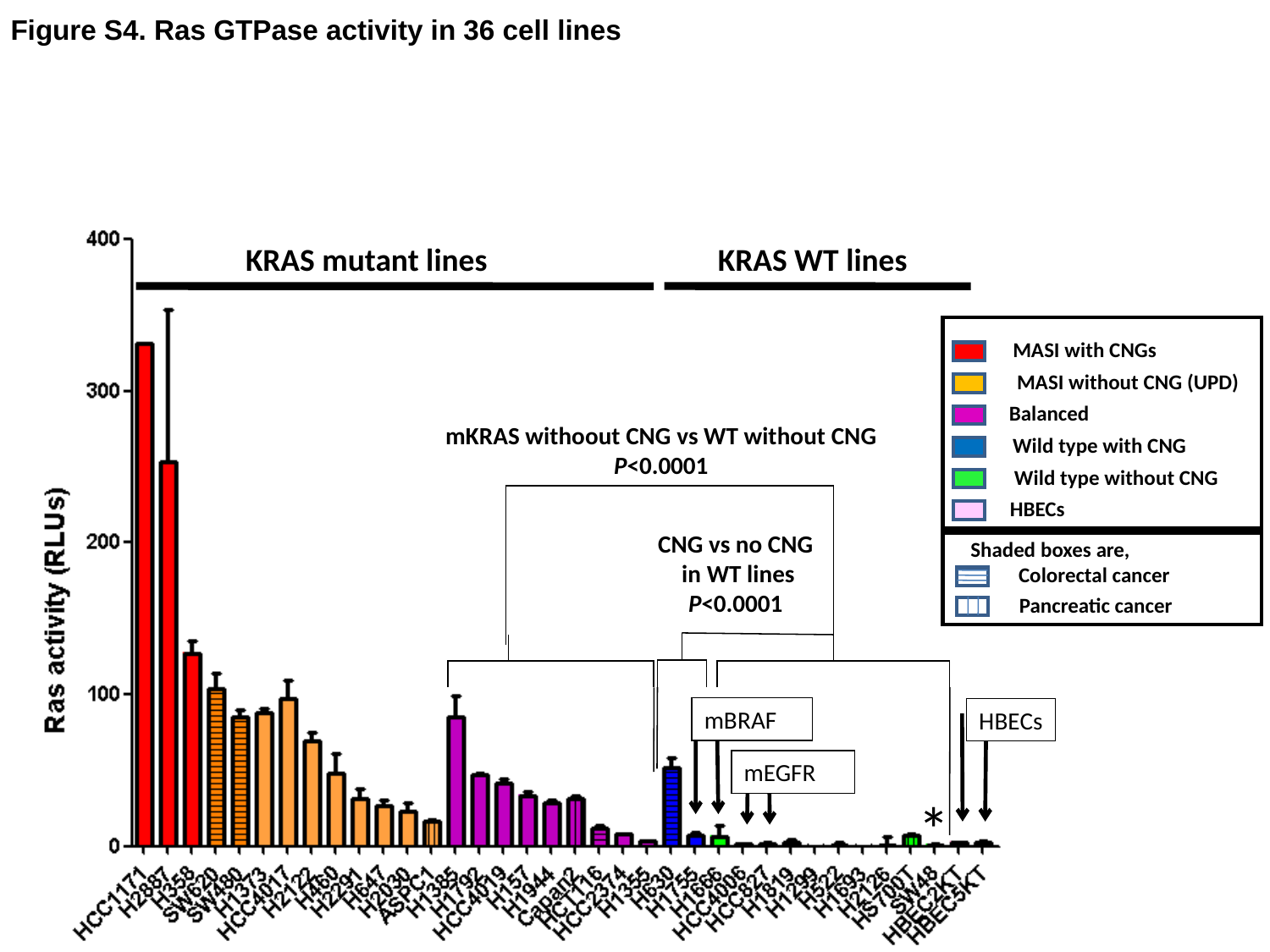

# Figure S4. Ras GTPase activity in 36 cell lines
KRAS mutant lines
KRAS WT lines
MASI with CNGs
MASI without CNG (UPD)
Balanced
Wild type with CNG
Wild type without CNG
HBECs
Shaded boxes are,
Colorectal cancer
Pancreatic cancer
mKRAS withoout CNG vs WT without CNG
P<0.0001
CNG vs no CNG
 in WT lines
P<0.0001
mBRAF
HBECs
mEGFR
*
